# The SARS-CoV-2 first wave impact in the acute inflammatory surgical pathologies

**DOI:** 10.1038/s41598-021-98878-w

**Published:** 2021-10-04

**Authors:** H. Guadalajara, J. L. Muñoz de Nova, M. Yiasemidou, M. Recarte Rico, L. D. Juez, J. García Septiem, P. Galindo Jara, M. García Virosta, E. Lobo Martínez, E. Martín-Pérez, S. Fernandez Gonzalez, O. Lopez-Fernandez, D. García-Olmo, PIACO Collaboration Group, J. M. Fernández-Cebrián, J. M. Jover, D. Acín-Gándara, E. Perea-del-Pozo, S. Dios-Barbeito, E. Martin-Antona, M. Durán-Poveda, B. Peinado Iribar, I. Pascual Migueláñez, S. Gortázar de las Casas, D. Fernández Luengas, A. Garcia Chiloeches, A. Puerta, E. Martín-Pérez, Y. García del Álamo Hernández, R. Maqueda González, R. Lathan, M. Gutiérrez Samaniego, L. Colao García, S. Núñez O’Sullivan, M. A. Vaquero, A. Picardo Nieto, A. Blazquez Martin, C. Vera-Mansilla, S. Soto Schüte, A. Gutiérrez Calvo, A. Sanchez Argüeso, S. Hernández-Villafranca, S. Qian Zhang, J. Mínguez García, L. Casalduero García, M. A. Iparraguirre, M. Florez Gamarra, J. M. Arguello Andres, B. Tallon Iglesias, F. Pereira Perez, D. Aparicio-Sanchez, V. Durán-Muñoz-Cruzado, F. Pareja-Ciuró, O. Cano-Valderrama, A. J. Torres-Garcia, L. Zarain Obrador, A. Moreno, M. A. Garcia Ureña, G. Paseiro, M. L. Fuenmayor-Valera, R. Pardo

**Affiliations:** 1 Department of General and Digestive Surgery, Fundación Jiménez Díaz University Hospital, Avda. Reyes Católicos 2, 28040 Madrid, Spain; 2 Department of General and Digestive Surgery, La Princesa University Hospital, Instituto de Investigación Sanitaria Princesa (IIS-IP), Madrid, Spain; 3 ST7 Colorectal Surgery, Bradford Teaching Hospitals, NIHR Academic Clinical Lecturer in General Surgery, University of Hull, Hull, UK; 4 Department of General and Digestive Surgery, Tajo University Hospital, Madrid, Spain; 5 Department of General and Digestive Surgery, Ramon y Cajal University Hospital, Madrid, Spain; 6 Department of General and Digestive Surgery, Torrejon University Hospital, Madrid, Spain; 7 Department of General and Digestive Surgery, Infanta Sofia University Hospital, Madrid, Spain; 8 Department of General and Digestive Surgery, Getafe University Hospital, Madrid, Spain; 9 Department of General and Digestive Surgery, Fuenlabrada University Hospital, Madrid, Spain; 10 Department of General and Digestive Surgery, Virgen del Rocio University Hospital, Sevilla, Spain; 11 Department of General and Digestive Surgery, Clínico San Carlos University Hospital, Madrid, Spain; 12 Department of General and Digestive Surgery, Rey Juan Carlos University Hospital, Madrid, Spain; 13 Department of General and Digestive Surgery, Quiron Madrid University Hospital, Madrid, Spain; 14 Department of General and Digestive Surgery, La Paz University Hospital, Madrid, Spain; 15 Department of General and Digestive Surgery, Principe de Asturias University Hospital, Madrid, Spain; 16 Department of General and Digestive Surgery, Sanitas la Moraleja University Hospital, Madrid, Spain; 17 Department of General and Digestive Surgery, Henares University Hospital, Madrid, Spain; 18 Department of General and Digestive Surgery, Infanta Leonor University Hospital, Madrid, Spain

**Keywords:** Health care, Diagnosis, Health services, Public health

## Abstract

Anecdotal evidence suggests that community infection control measures during the COVID-19 outbreak have modified the number and natural history of acute surgical inflammatory processes (ASIP—appendicitis, cholecystitis, diverticulitis and perianal abscesses) admissions. This study aims to evaluate the impact of the COVID-19 pandemic on the presentation and treatment ASIP and quantify the effect of COVID-19 infection on the outcomes of ASIP patients. This was a multicentre, comparative study, whereby ASIP cases from 2019, 2020 and 2021 (March 14th to May 2nd) were analyzed. Data regarding patient and disease characteristics as well as outcomes, were collected from sixteen centres in Madrid, and one in Seville (Spain). The number of patients treated for ASIP in 2019 was 822 compared to 521 in 2020 and 835 in 2021. This 1/3rd reduction occurs mainly in patients with mild cases, while the number of severe cases was similar. Surgical standards suffered a step back during the first wave: Lower laparoscopic approach and longer length of stay. We also found a more conservative approach to the patients this year, non-justified by clinical circumstances. Luckily these standards improved again in 2021. The positive COVID-19 status itself did not have a direct impact on mortality. Strikingly, none of the 33 surgically treated COVID positive patients during both years died postoperatively. This is an interesting finding which, if confirmed through future research with a larger sample size of COVID-19 positive patients, can expedite the recovery phase of acute surgical services.

## Introduction

The first case of SARS-CoV-2 (COVID19) in Spain was confirmed on January 31st 2020. Since then, the virus has spread throughout Spain, making it one of the most affected countries in the world, with the Community of Madrid being the most intensely impacted. In addition to the lifestyle changes, a rapid restructuring and reorganisation of healthcare practices took place. In many countries, the COVID-19 crisis found the healthcare systems unprepared and precipitated a chain of events aiming to ensure that hospital services were not overwhelmed. Surgery services were greatly re-structured during the counter-pandemic measures, reducing surgeries only to urgent and emergency cases^1^.

The pandemic had an inevitable impact on the care provided to acute surgical patients. Guided by early reports of worryingly high adverse events rates after surgery, conservative approaches have been favoured^2,3^. Attendances at the emergency departments due to surgical pathologies were reduced significantly^4,5^. This phenomenon could suggest a change in the epidemiological presentation of these pathologies during the COVID-19 pandemic or merely the avoidance of seeking medical attention at all costs, due to fear of contracting the virus. Understanding the underlying reasons for the altered presentation patterns and the impact on patient outcomes within the context of a global pandemic, is crucial for guiding treatment during the recovery phase or a second wave of COVID-19.

From the beginning of the pandemic until the end of the study, four waves hit Spain. Although COVID-19 incidence remained similar during each wave, severity decreased, largely due to the administration of the SARS-CoV-2 vaccine. Nonetheless, anecdotal evidence suggest that surgical practice continues to remain altered to the current day.

The aim of this study was to evaluate the characteristics of the patients with appendicitis, cholecystitis, diverticulitis or perianal abscesses (Acute Surgical Inflammatory Processes—ASIP) comparing the same 2019, 2020 and 2021 timeframe (March 14th to May 2nd). We also tried to identify factors that could be related with the differences observed within the three periods.

## Methods

### Study design

This was a multicentre comparative study, carried out at sixteen hospitals of the Community of Madrid and one hospital in Seville (Andalusia, Spain). Fourteen hospitals belong to the Madrid Public Healthcare System and serve an estimate of 4,400,000 inhabitants, 66.4% of Community of Madrid population.

### Study population and time frame

Consecutive patients older than eighteen years of age with an ASIP diagnosis (acute appendicitis, acute cholecystitis, acute diverticulitis or perianal abscess) who presented to hospital from March 14th 2020 (date of the declaration of the state of alarm by Spanish Government) to May 2nd 2020 (beginning of the gradual de-escalation plan) were included in this study. Patients treated during the same time frame in 2019 and 2021 were also included. Patients were excluded if an ASIP had been diagnosed within 30 days before admission or if the admission was due to a scheduled surgery for definitive treatment of the ASIP.

Demographic data were also collected, these included the Charlson Comorbidity Index (CCI)^6^, ASIP diagnosis and severity (Table 1), COVID-19 diagnosis, treatment modality and mortality/morbidity (Clavien-Dindo Classification)^7^. Diverticulitis and cholecystitis severity were assessed according to the modified Hinchey classification^8^ and the Tokyo guidelines 2018^9^, respectively. Complications were considered severe in cases of grades 3 to 5 of Clavien-Dindo Classification and mild in grades 1 and 2.

**Table 1:** Severity classification of acute surgical inflammatory processes.

	Mild	Moderate	Severe
Appendicitis	Phlegmonous	Gangrenous non-perforated Appendicular phlegmon	Perforated
Cholecystitis	Grade 1^a^	Grade 2^a^	Grade 3^a^
Diverticulitis	Grade 1^b^	Grade 2^b^	Grades 3-4^b^
Perianal abscess	Unilateral	Bilateral	Fournier’s gangrene

^a^Modified Hinchey Classification^8^.

^b^Tokyo Guidelines 2018^9^.

### Statistical analysis

Categorical variables were summarized as counts and proportions. Chi-squared Pearson test or Fisher's exact test were used for all relevant comparisons.

After confirming than the continuous variables did not follow a normal distribution using the Shapiro–Wilk test, they were summarized as median and interquartile range, using Kruskal–Wallis test for comparison purposes. Only when the result was significant, multiple comparisons were subsequently performed, adjusting its significance by the Bonferroni method. The comparison of ASIP and COVID cases was made with the Pearson correlation test. When appropriate, logistic regression analysis was used to identify factors independently associated with those variables that showed differences with the 2019 group. The variables were included in the model when the p-value was less than 0.1 on the univariate analysis or due to clinical importance. All reported p-values were 2-sided, and p-values < 0.05 were considered statistically significant. All analyses were performed with SPSS software (Version 20.0, Chicago, IL, USA).

### Ethical considerations

Ethical approval for this study was granted by the Clinical Research Ethics Committee of Hospital Universitario de La Princesa, Madrid, Spain (approval number: 2020–4076). This study was carried out according to the principles of the Good Clinical Practice as defined in the International Conference on Harmonisation (ICH) and in full conformity with ethical and deontological rules. The Spanish and European regulations of protection of personal data were also observed during the study. For this retrospective study, the need for informed consent is waived by the Clinical Research Ethics Committee of Hospital Universitario de La Princesa, Madrid, Spain.

### Consent for publication

Not applicable.

## Results

While in 2019 a total 822 patients were treated for ASIP, these numbers decreased to 521 in 2020 (-36.6%) and rose again in 2021 to 835 patients. In 2019 and 2020, the median age was 49 years, but in 2021 was slightly lower with 47 years, with statistical differences. Male gender was predominant during the years analysed (456 in 2019, 55.5%; 316 in 2020, 60.8%; and 497 in 2021, 58.1%). Appendicitis was the most frequent diagnosis (43.5% in 2019, 45.9% in 2020 and 42.8% in 2021), followed by cholecystitis in 2020 and 2021 (25.1% and 25.7%, respectively) and perianal abscesses in 2019 (21.7%). The proportion of severe cases at diagnosis was higher in 2020 than in 2019 (16.1% vs. 9.9%; *p* < 0.001) and in 2021 (16.1% vs. 7.3%; *p* < 0.001). This proportion was slightly lower in 2021 than in 2019 (9.9% vs. 7.3%; p = 0,056). The absolute number of patients with severe cases were 81 in 2019, 84 in 2020 and 62 in 2021. Mortality was very similar among the three years: 7 cases in 2019 (0.9%), 5 cases in 2020 (1%) and 8 cases in 2021 (0.9%).

In the patients treated during 2020 and 2021, COVID infection was related to a higher rate of severe complications (19.0% vs. 5.2%; p < 0.001), but no rise the risk of mortality significantly (3.4% vs. 1%; p = 0.144). Compared to 2020, COVID positive patients were more commonly surgically treated in 2021 (80.0% vs. 45.9%; p = 0.028). This increase in surgical treatment in COVID positive patients during 2021 didn't associate an increase in severe complications (17.6% in 2020 vs. 12.5% in 2021; p = 1). Strikingly, none of the 33 surgically treated COVID positive patients during both years died postoperatively.

**Figure 1:**
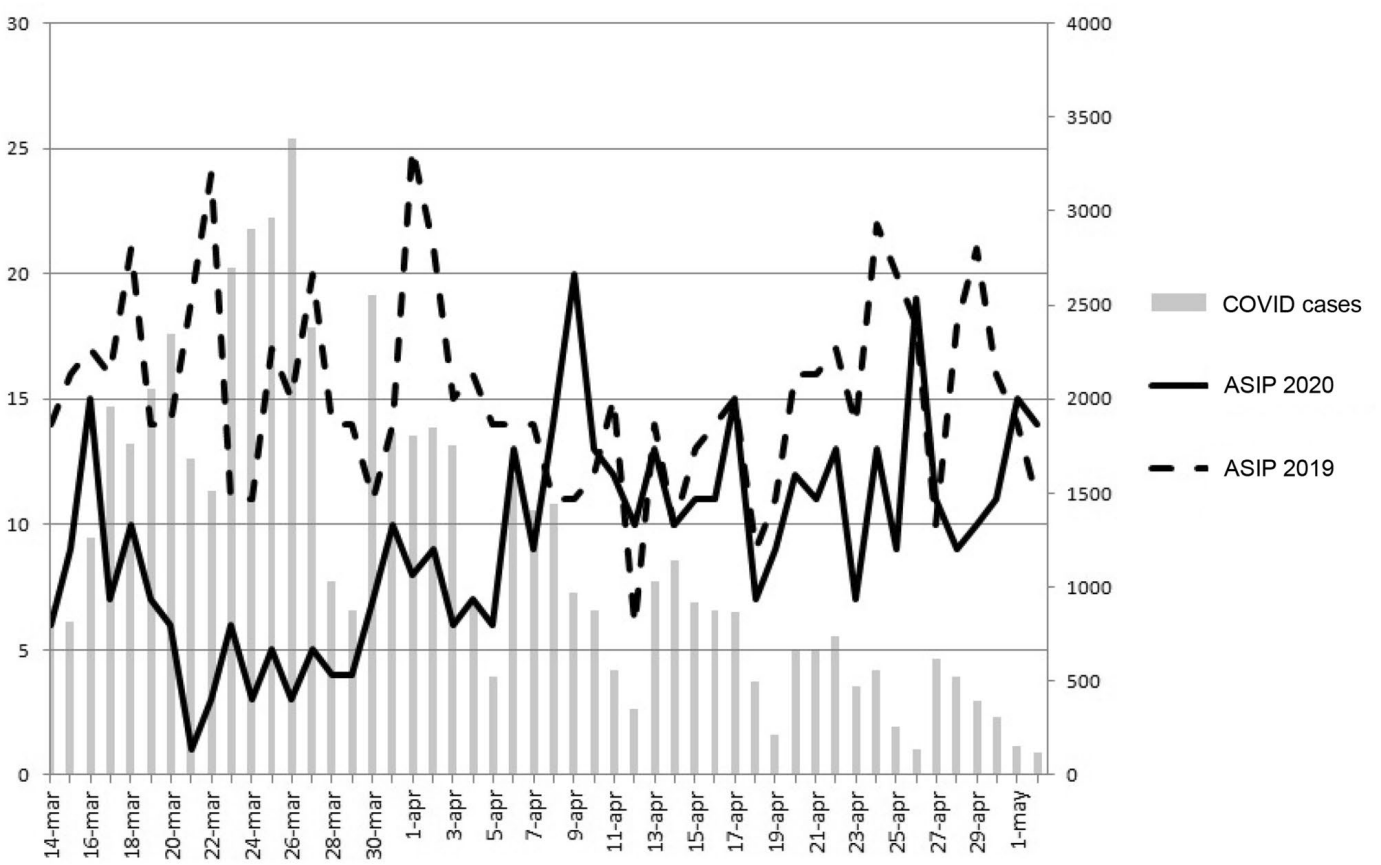
Study timeline comparing daily number of patients of 2020 with 2019 and the number of COVID-19 cases diagnosed each day (This data refers to the Community of Madrid only). *COVID-19* coronavirus disease 2019.

**Figure 2:**
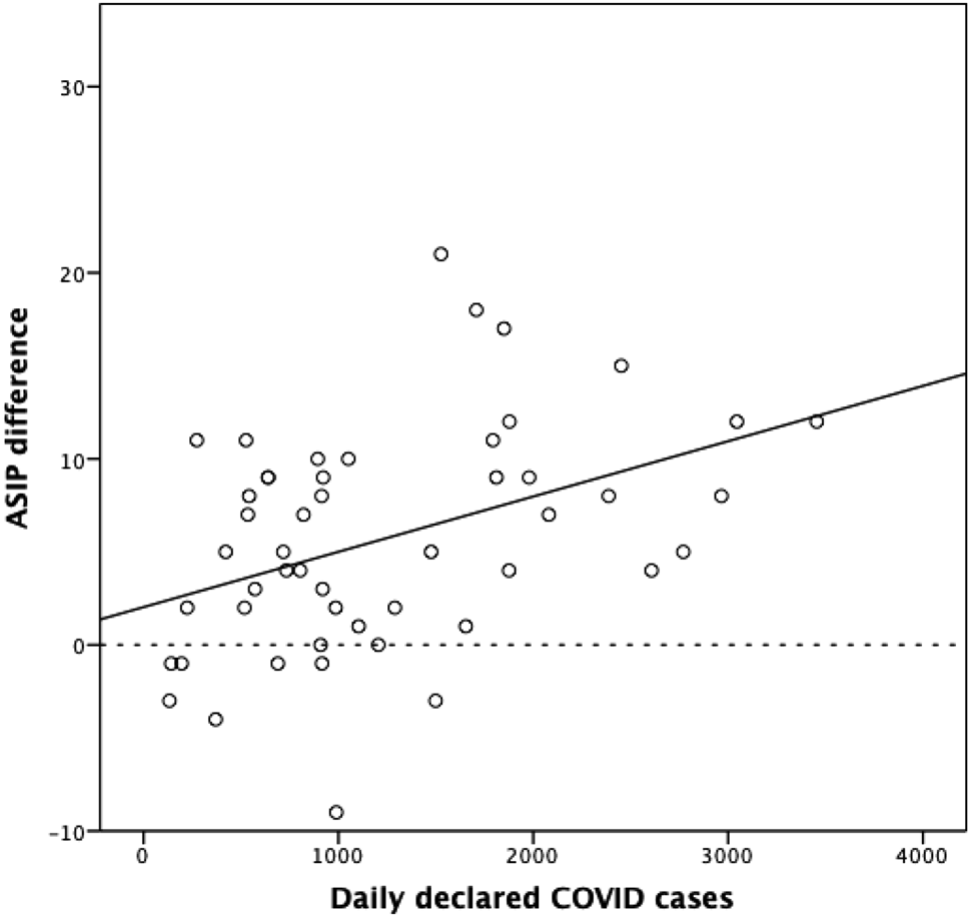
Dispersion diagram of daily difference in number of ASIP within 2019 and 2020 and the number of COVID-19 cases (Community of Madrid data only). *ASIP* appendicitis, cholecystitis, diverticulitis and perianal abscesses, *COVID-19* coronavirus disease 2019.

**Figure 3:**
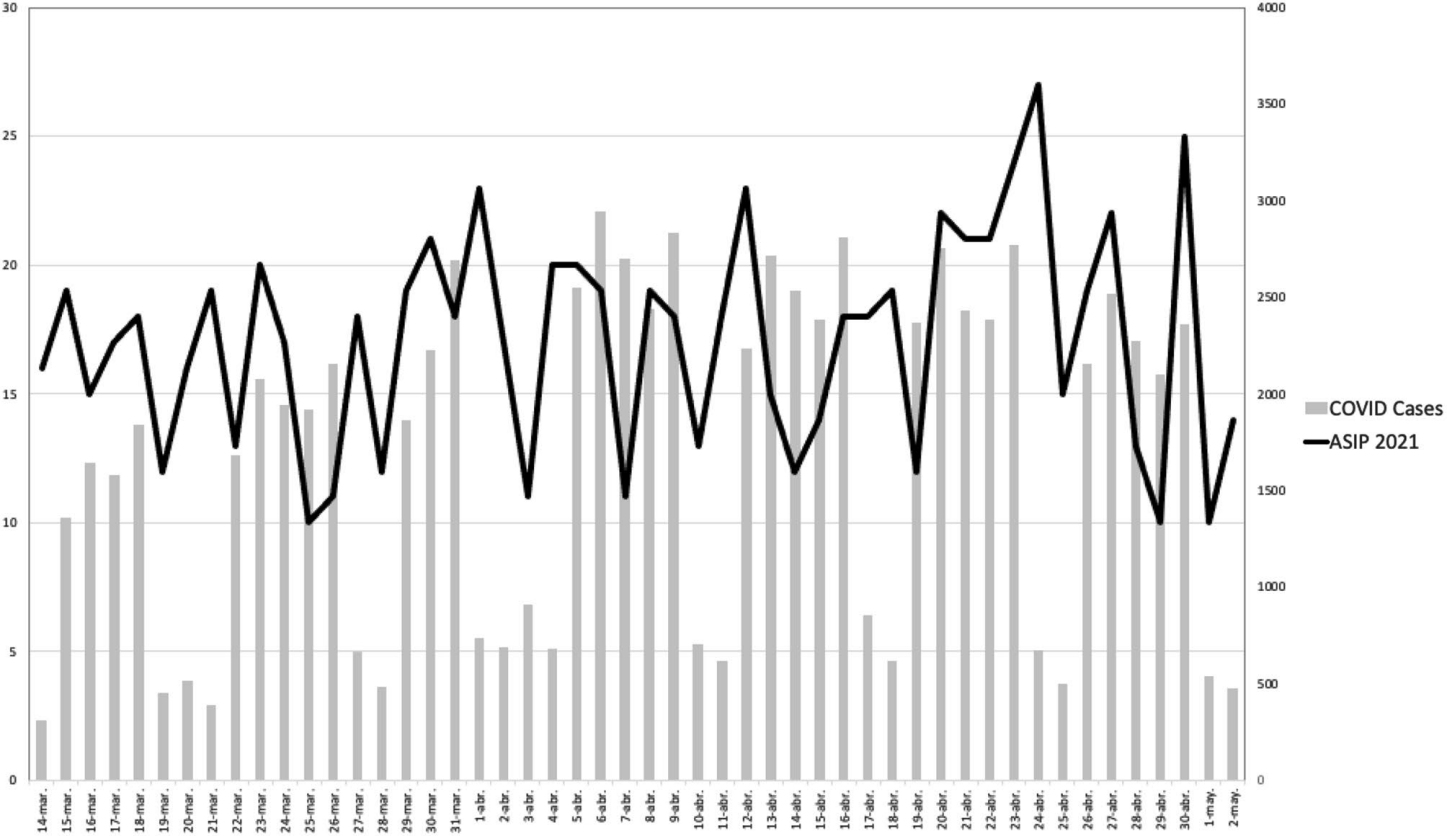
Relation between daily COVID and ASIP cases in 2021.

Figure 1 displays the number of cases (2020 group), controls (2019 group) and COVID-19 cases per day. We found a moderate correlation between the difference in cases among the two years and the number of declared cases of COVID (Pearson correlation coefficient = 0.413; *p* = 0.003) (Fig. 2). COVID and ASIP cases remained stable along the 2021 (Fig. 3).

### Appendicitis

In 2020, 239 patients were treated for appendicitis, which represents a reduction of 33.4% from the previous year (359 patients), returning in 2021 to a number very similar to 2019 (366). This reduction occurs mainly in cases of mild severity (227 in 2019, 116 in 2020 [− 48.7%] and 223 in 2021 [− 1.76%]). Thirteen out of 148 patients with appendicitis tested for COVID-19 in 2020 were positive (8.8%). In 2021, only 8 of the 343 patients tested were positive (2.4%). While all patients treated in 2021 were tested, only 66.1% of patients treated in 2020 were tested. Compared to 2019, a significantly higher number of patients exhibit delayed presentation (> 7 days after onset of symptoms) during 2020 (7.9% vs. 2.2%; *p* = 0.001) and 2021 (5.8% vs. 2.2%; *p* = 0.016). Surgical treatment was employed in the 2020 group less frequently than in 2019 (92.5% vs. 97.5%; *p* = 0.007) and 2021 (92.5% vs. 97.5%; *p* = 0.006). Additionally, the laparoscopic approach was less frequently adopted in 2020 than in 2019 and 2021, and more used in 2021 than in 2019. Post-treatment complications were more frequent in the 2020 group (23%), however the number of severe complications was similar between the three groups (11, 14 and 12). There was no mortality in these patients (Table 2).

**Table 2:** Appendicitis patients characteristics.

	Year			p-value		
	2019	2020	2021	2019 vs. 2020	2020 vs. 2021	2019 vs. 2021
Male gender n (%)	175 (48.7)	138 (57.7)	206 (56.3)	**0.038**	0.787	0,050
Age (years) median (IQR)	37 (27–51)	39 (27–54)	36 (24–49)	0.076*		
CCI median (IQR)	0 (0–1)	0 (0–1)	0 (0–1)	0.203*		
Delay > 7 days n (%)	8 (2.2)	19 (7.9)	21 (5.8)	**0.002**	0.381	**0.026**
**Severity at diagnosis**						
Mild n (%)	228 (63.4)	116 (49.6)	223 (60.9)	**0.003**	**0.001**	0.125
Moderate n (%)	81 (22.6)	68 (29.1)	104 (28.4)			
Severe n (%)	50 (14.0)	50 (21.4)	39 (10.7)			
Appendicular lump n (%)	23 (6.4)	26 (10.9)	22 (6.0)	0.072	**0.044**	0.947
Surgical treatment n (%)	350 (97.5)	221 (92.5)	357 (97.5)	**0.007**	**0.006**	1
Laparoscopic approach n (%)	324 (92.6)	183 (83.2)	345 (96.6)	**0.001**	** < 0.001**	**0.026**
Length of stay (days) median (IQR)	2 (1–4)	2 (1–5)	1 (1–3)	0.189	** < 0.001**	**0.022**
Complications (any grade) n (%)	37 (10.3)	55 (23)	41 (11.2)	** < 0.001**	** < 0.001**	0.788
Severe complications (grade 3–5) n (%)	11 (3.1)	14 (5.9)	12 (3.3)	0.143	0.185	1

*CCI* Charlson Comorbidity Index.

*Non-significant p-value in Kruskal–Wallis test, therefore no multiple comparations were performed.

The median Charlson Comorbidity Index (CCI) of patients treated surgically in 2020 were significantly higher than in 2019 (1 vs. 0; *p* = 0.041]). During pandemic period (2020 and 2021), surgical treatment was less commonly employed for patients with COVID-19 (76.2% vs. 96.4%; *p* < 0.001). The logistic regression model analysis showed than a delay longer than 7 days (OR 0,028; CI95% 0.010–0.080) and COVID-19 infection (OR 0.091; CI95% 0.022–0.388) were the only predictor factors for conservative treatment. Similarly, during the pandemic period the COVID-19 infection was related to employing an open (versus laparoscopic) approach (31.3% vs. 5.8%; *p* = 0.002). However, only severe cases at diagnosis were related with a higher risk of severe complications (39.1% vs.11.4%; *p* = 0.001).

### Cholecystitis

A reduction in the number of cholecystitis cases was also observed during 2020 (131 patients vs.170 patients in 2019 [− 22.9%] and 220 in 2021); similarly to appendicitis, mild severity cases showed the greatest reduction (− 35.7%) compared to 2019. The number of severe cases were similar for both years (17 in 2019 and 2020), and slightly lower in 2021 (12 patients). Thirteen of the 103 patients tested (12.6%) in 2020 and 3 of 187 (1.6%) in 2021, were positive for COVID-19. The percentage of patients treated conservatively during 2020 (68.7%) was more than double compared to 2019 (32.6%) and 2021 (31.2%). The laparoscopic approach remains the preferred technique in the three years (92.2%, 90.2% and 94.0%). Length of stay was longer in 2020 (6 days) compared to 2019 and 2021 (4 days both). No differences in the rate of severe complications was observed between the years analysed (Table 3).

**Table 3:** Cholecystitis patients characteristics.

	Year			p-value		
	2019	2020	2021	2019 vs. 2020	2020 vs. 2021	2019 vs. 2021
Male gender n (%)	102 (60)	76 (58.5)	115 (52.3)	0.881	0.311	0.155
Age (years) median (IQR)	66 (54.7–78.2)	70 (53–79)	64 (48–79)	0.422*		
CCI median (IQR)	3 (1–5)	3 (1–5)	2 (1–5)	0.327*		
Delay > 7 days n (%)	22 (12.9)	22 (16.8)	24 (11.5)	0.439	0.219	0.784
**Severity at diagnosis**						
Mild n (%)	70 (41.4)	45 (34.6)	107 (48.9)	0.431	**0.006**	0.135
Moderate n (%)	82 (48.5)	68 (52.3)	100 (45.7)			
Severe n (%)	17 (10.1)	17 (13.1)	12 (5.5)			
Surgical treatment n (%)	115 (67.6)	41 (31.3)	150 (68.8)	** < 0.001**	** < 0.001**	0.894
Laparoscopic approach n (%)	106 (92.2)	37 (90.2)	141 (94.0)	0.745	0.482	0.734
Length of stay (days) median (IQR)	4 (3–7.2)	6 (4–10)	4 (2–7)	**0.002**	** < 0.001**	0.821
Complications (any grade) n (%)	28 (16.5)	33 (25.2)	33 (15.0)	0.085	**0.026**	0.798
Severe complications (grade 3–5) n (%)	14 (8.2)	10 (7.6)	11 (5.0)	1	0.439	0.278
Mortality n (%)	3 (1.8)	5 (3.8)	4 (1.8)	0.301	0.303	1

*CCI* Charlson Comorbidity Index.

*Non-significant p-value in Kruskal–Wallis test, therefore no multiple comparations were performed.

In 2020, COVID-19 infection was related with the election of conservative treatment (100% vs. 65.6%; *p* = 0.009), but this relation it wasn’t observed during 2021 (50% vs. 30.4%; p = 1). During the years 2020 and 2021, age (conservative/surgical treatment group: 74,5 vs 59 years; *p* < 0.001) and CCI (4 vs. 1; *p* < 0.001) were also related to conservative treatment. In the multivariate analysis CCI (OR 0.687; IC95% 0.612–0.771) and COVID infection (OR 0.043; CI95% 0.005–0.362) were both independently related with the conservative treatment.

Although age (*p* < 0.001), CCI (*p* < 0.001), COVID infection (6.5 vs. 5 days; *p* = 0.006) and non-surgical treatment (7 vs. 3 days; *p* < 0.001) demonstrated a statistical significance initially on univariate analysis, none of them were significant on multivariable analysis.

### Diverticulitis

Patients with diverticulitis had the largest decrease in hospital admissions during 2020 (46 cases compared to 115 in 2019 and 96 in 2021). Whist, mild cases were dramatically reduced (− 69.6%), severe cases were increased (6 in 2019 and 11 in 2020). Five of thirty-four patients (14.7%) with diverticulitis tested for COVID-19, were found to be positive during 2020. Only 5 of 82 patients tested during 2021 were positive (6.1%). The percentage of patients treated surgically was greater in 2020 (30.4%) than in 2019 (13.9%; p = 0.027) and 2021 (15.6%; p = 0.068). Compared to the previous year, patients treated during 2020 had a longer length of stay, as well as a higher rate of overall and severe complications (Table 4).

**Table 4:** Diverticulitis patients characteristics.

	Year			p-value		
	2019	2020	2021	2019 vs. 2020	2020 vs. 2021	2019 vs. 2021
Male gender n (%)	54 (47.0)	21 (45.7)	49 (51.0)	1	0.673	0.651
Age (years) median (IQR)	66 (54–76)	62 (48.7–71.7)	61.5 (49–76.5)	0.430*		
CCI median (IQR)	2 (1–4)	2 (0–3)	2 (1–4)	0.490*		
Delay > 7 days n (%)	11 (9.6)	9 (19.6)	19 (20.7)	0.146	1	**0.043**
**Severity at diagnosis**						
Mild n (%)	92 (80.7)	28 (68.3)	70 (72.9)	** < 0.001**	**0.004**	0.394
Moderate n (%)	16 (14.0)	2 (4.9)	18 (18.8)			
Severe n (%)	6 (5.3)	11 (26.8)	8 (8.3)			
Surgical treatment n (%)	16 (13.9)	14 (30.4)	15 (15.6)	**0.027**	0.068	0.877
Laparoscopic approach n (%)	8 (53.3)	3 (21.4)	7 (46.7)	0.166	0.245	1
Length of stay (days) median (IQR)	5 (3–8)	7 (5–12)	6 (3–10)	**0.008**	0.084	1
Complications (any grade) n (%)	15 (13.0)	17 (37.0)	17 (17.7)	**0.001**	**0.021**	0.454
Severe complications (grade 3–5) n (%)	5 (4.3)	7 (15.2)	7 (7.3)	**0.040**	0.146	0.535
Mortality n (%)	1 (0.9)	0 (0)	1 (1.0)	1	1	1

*CCI* Charlson Comorbidity Index.

*Non-significant p-value in Kruskal–Wallis test, therefore no multiple comparations were performed.

Severity at time of diagnosis was related with opting for surgical management (84,6% in severe cases vs. 10.0% in non-severe cases; *p* < 0.001). Severe cases (Hinchey 3 and 4) were more frequent in COVID positive patients (50.0% vs. 11.3%; p = 0.006). Higher CCI (p = 0.046), COVID infection (p = 0.019) and surgical treatment (p < 0.001) were related with longer length of stay, but none of the reach a statistical significance on multivariate analysis. When complications were used as a dependent variable for analysis, surgical treatment was the only predictor of severe complications (OR 15.799; CI95% 3.838–65.028) during the pandemic years.

### Perianal abscesses

Similar to diverticulitis, perianal abscesses experienced a significant reduction during 2020 (105 cases) compared to 2019 (178) and 2021 (173). Six out of 60 patients (10%) tested during 2020 were positive for COVID-19, as well as 5 of the 156 tested in 2021. The rate of patients than wait more than 7 days before seeking specialised care was significant higher in 2020 compared to 2019 (27.6% vs. 15.3%; p = 0.018). No differences were found between the two groups in the other variables analysed and resumed in Table 5.

**Table 5:** Perianal abscess patients characteristics.

	Year			p-value		
	2019	2020	2021	2019 vs. 2020	2020 vs. 2021	2019 vs. 2021
Male gender n (%)	125 (70.2)	81 (77.1)	127 (73.4)	0.261	0.581	0.586
Age (years) median (IQR)	46.5 (37–59)	45 (35–59.5)	48 (36–63)	0.588*		
CCI median (IQR)	0 (0–2)	0 (0–2)	1 (0–2)	0.088*		
Delay > 7 days n (%)	27 (15.3)	29 (27.6)	34 (20.2)	**0.018**	0.207	0.284
**Severity at diagnosis**						
Mild n (%)	149 (85.1)	87 (83.7)	151 (88.3)	0.825	0.436	0.322
Moderate n (%)	18 (10.3)	13 (12.5)	17 (9.9)			
Severe n (%)	8 (4.6)	4 (3.8)	3 (1.8)			
Surgical treatment n (%)	177 (99.4)	104 (99.0)	171 (98.8)	1	1	0.619
Length of stay (days) median (IQR)	1 (1–3)	1 (1–3)	1 (1–2)	0.388*		
Complications (any grade) n (%)	21 (11.8)	12 (11.4)	17 (9.8)	1	0.825	0.673
Severe complications (grade 3–5) n (%)	15 (8.4)	6 (5.7)	9 (5.2)	0.544	1	0.325
Mortality n (%)	3 (1.7)	0 (0)	3 (1.7)	0.297	0.292	1

*CCI* Charlson Comorbidity Index.

*Non-significant p-value in Kruskal–Wallis test, therefore no multiple comparations were performed.

## Discussion

Our study represents an extensive observational registry of consecutive patients treated for ASIP during one of the most challenging periods of the COVID-19 pandemic in a severely affected area.

COVID-19 positive patients did not experience an increased risk of mortality events. Strikingly, none of the 33 surgically treated COVID positive patients during both years died postoperatively. This result should be interpreted with caution due to the limited number of COVID-19 positive patients included in this study. However, recently published studies have found evidence that points in this same direction^10,11^.

The effect of the COVID pandemic on emergency surgery has been a subject of analysis in several studies^12–16^. The results of the PIACO study are consistent with other studies in the current literature. The significant decrease in ASIP admission during COVID-19 pandemic was also demonstrated in a matched case–control study of appendicitis^17^. Our data also reflect that the most dramatic drop in admissions occurred during the peak of the pandemic and tends to normalize with the decrease in the number of confirmed COVID-19 cases. This is more evident during the 2021 period.

Whilst the number of admissions was reduced, the absolute number of severe cases during the pandemic was similar to 2019. The relative increase of the complex cases may be explained by the reduction of less severe cases. In the appendicitis subgroup, the absolute number of severe cases was similar between the two years, however, the overall number of complications was significantly higher in the 2020 group. There was no difference in severe complications between the two groups.

The exact reason for the drop in admission numbers is unknown and it is an interesting field of research. Perhaps the most plausible theory, is that low complexity cases were treated as outpatients, self-medicated or even cured without any treatment. This could be especially relevant in mild diverticulitis cases, which display the highest case reduction (60%), and appendicitis. Outpatient treatment of mild diverticulitis in low-risk patients was already a routine practice in most of the participant hospitals and recent guidelines recommend that antibiotics for uncomplicated diverticulitis, are not necessary^18^. However, current data supports that this concept could be translated to non-complicated appendicitis and is an interesting field for future research.

The decline in surgical admission numbers can also be explained by self-medication with over the counter medication. It has been proven that lockdown helped to stop the expansion of the coronavirus^19^ and Spanish population suffered one of the hardest confinements imposed by the Spanish government. As the message sent to the population was to avoid leaving their houses except for essential activities, it is possible that patients with mild symptoms tried not to seek medical attention. This behavioural modification combined with the fear of getting exposed to COVID-19 at the hospital could also contribute to the reduction of ASIP admissions.

The findings of the study suggest that all ASIP except for diverticulitis have an increased evolution time. This would explain why despite late presentation of patients the severity of ASIP cases (with the exception of diverticulitis) during the pandemic was similar to last year. However, due to the paucity of evidence on how ASIP cases progress without any medical attention, it is difficult to prove a causative relationship between the prolonged natural history of a disease and the lack of a higher number of severe presentations in our cohort. Other factors may also have contributed to the observed reduction in cases. There is some evidence that air pollution could be related to inflammatory gastrointestinal pathologies. This association has a plausible explanation relating inhaled pollutants being ingested after mucociliary clearance. Pollutants can then affect intestinal epithelium and microbiota altering lipid metabolism and particularly intestinal redox lipids, which are associated with intestinal and systemic inflammation^20^. At an epidemiological level, this relation has been observed especially regarding acute appendicitis, in which a seasonal pattern has been identified^21^, and some studies relate this seasonality to the level of air pollution^22–25^. COVID-19 pandemic caused a series of lockdown measures on most of the countries it affected that led to a great reduction in personal mobility, goods shipping and industry production. Along with the reduction in transportation, there has been an air pollution reduction observed in Spain^26^.

COVID-19 infections clearly play a role in the host immune response mainly through modifying cytokine production resulting in pulmonary tissue damage and the immune insufficiency that may increase viral replication^27^. Coronavirus also produces lymphopenia in 82,1% of the hosts^28^. These coronavirus-host interactions may also have influenced the response to acute inflammatory surgical diseases mitigating the immunological response and thus prolonging or stopping the natural history of ASIP cases.

It is not possible to reduce emergency surgery without altering the quality of care provided to surgical patients^29^. The COVID-19 pandemic has impacted our practice^3,30^ in favour of more conservative attitudes^2^. This was shown in this study as well. Conservative treatment was safe and did not increase the overall morbidity. However, this conservative approach impeded the patients to benefit from the advantages of laparoscopic appendicectomy and early cholecystectomy in acute cholecystitis. Laparoscopy rates, length of stay, and conservative management rates were recovered in the last period analyzed.

During the COVID-19 crisis postponement of elective procedures was supported by esteemed surgical societies^31–33^. Delay is not usually an option with emergency cases; the dilemma is between surgical or conservative treatment. In these situations, we have seen a change to a more conservative approach, aiming to spare patients the adverse events after surgery which were reported at the early days of the COVID-19 pandemic. This is more prominent in the treatment of cholecystitis, whereby we found a complete inversion in the percentages of surgical versus conservative treatment, which was not associated with a variance in patient characteristics. As a result of the selection of conservative treatment, the length of stay of these patients has also risen significantly. Moreover, fewer procedures were performed laparoscopically, which may also have contributed to the prolonged length of stay. This phenomenon can be attributed to concerns about a higher risk of transmissions due to aerosolization. However, more recent literature does not support a higher risk of transmission with smoke^34^ and some authors publish that laparoscopy may be safer^35^.

Whilst some centres may offer antibiotics as treatment for uncomplicated appendicitis^36,37^ a recent multicentre randomised controlled trial demonstrated lack of non inferiority in efficacy of treatment with antibiotics^38^. Of 273 patients randomised to the surgical group, all but 1 underwent successful appendectomy (success rate of 99.6% (95% CI 98.0–100.0%)). Conversely, in the antibiotic group, 70 of 257 patients (27.3%; 95% CI 22.0–33.2%) required appendectomy within one year from the initial presentation^38^. Similarly, Salinell et al.^37^, in a 2016 meta-analysis showed recurrence of appendicitis in 22.6% of patients treated with antibiotics within one year. On the other hand, complications were higher for the group that underwent surgery^37^. It should be noted that both studies refer to uncomplicated appendicitis^37,38^. Overall, the role of the physician in treating any pathology is to provide the patient an informed decision, explaining their options and risks and benefits. The authors of this study cannot advocate in favour of any of the two treatments based on current evidence.

Nonetheless, this study has some limitations. It only included patients who required admission in a hospital setting. Information about outpatients is not available, which could represent a selection bias. However, this study provides useful insight on the presentation patterns of ASIP pathology at hospitals covering approximately the 70% of the population of the Community of Madrid. These reductions in mild cases without a rise in severe cases suggest that outpatient treatment could be useful in some instances. The generalisability of the results of this study is further enhanced by the inclusion of both public and private hospitals. Moreover, during the study period, primary care emergencies and elective services remained closed and the healthcare professionals were redeployed to other facilities caring for COVID patients (e.g., Hospital created in the Madrid Fair). In this setting, all the emergencies were treated in the hospitals. Surgeons were redeployed in emergency services in the hospitals looking after surgical emergencies. Therefore, the number of patients with ASIP diagnosis that were treated in community-based facilities (and hence not included in this study) is minimal, and no long-term effects have been studied. Moreover, although the overall sample size is adequate, the number of COVID-19 positive patients included in this study is limited; therefore, any outcomes related to this group of patients should be interpreted with caution.

In conclusion, our data show that the COVID-19 outbreak has changed the patterns of the presentation of ASIP cases, with a higher percentage of severe cases seeking medical attention. Surgical practice has been altered for appendicitis and cholecystitis, with a greater number of cases being treated conservatively. Data from 2021 however, demonstrate that the number of admissions have recovered and practice has largely retroverted to the pre-COVID era.

Whilst the COVID-19 outbreak selected more complex cases, the positive COVID-19 status itself did not have a direct impact on either morbidity or mortality. This is an interesting finding which, if confirmed through future research with a larger sample size of COVID-19 positive patients, can expedite the recovery phase of acute surgical services.

## Data Availability

The datasets used and/or analysed during the current study are available from the corresponding author on reasonable request.
